# No Iron in the Coloring Matter of the Blood

**Published:** 1875-10

**Authors:** 


No Iron in the Coloring Matter of the Blood.—More than a year ago MM. Paquelin and Jolly, in a communication to the French Academy of Sciences, asserted that "iron exists in the blood-globules in the state of tribasic ferros phosphate," but that "hæmatosin contains no iron, as M. Chevreul had already proved, observing that the composition of this coloring matter varies according to the nature of the solvent applied for its extraction, but at the same time declaring that we know of no process by which it may be obtained in a pure state." In a recent paper the same authors report: "We have at last been able to obtain the hæmatic pigment in a state of perfect purity and entirely free from iron. This hæmatosin exhibits the following properties: It burns, without leaving any ash, like resinous substances; it is insoluble in pure water; it dissolves a very little in ammonia, to which it gives a pale yellow tint; it is acted upon by caustic potash and soda, to which it communicates a brown tint, and it is slightly soluble in alcohol; the solution becomes amber-colored. The solvents of hæmatosin are ether, chloroform, benzine, and carbonic disulphide. With these bodies, the diluted solution is amber-colored; concentrated, it is red."—*Druggists' Cir.*

